# Ultrasound Tissue Characterization of Carotid Plaques Differs Between Patients with Type 1 Diabetes and Subjects without Diabetes

**DOI:** 10.3390/jcm8040424

**Published:** 2019-03-28

**Authors:** Esmeralda Castelblanco, Àngels Betriu, Marta Hernández, Minerva Granado-Casas, Emilio Ortega, Berta Soldevila, Anna Ramírez-Morros, Josep Franch-Nadal, Manel Puig-Domingo, Elvira Fernández, Angelo Avogaro, Núria Alonso, Dídac Mauricio

**Affiliations:** 1 Department of Endocrinology and Nutrition, University Hospital and Health Science Research Institute Germans Trias i Pujol, 08916 Badalona, Spain; esmeraldacas@gmail.com (E.C.); mgranado@igtp.cat (M.G.-C.); bsolde@hotmail.com (B.S.); aramirez@igtp.cat (A.R.-M.); mpuigd@gmail.com (M.P.-D.); 2 Center for Biomedical Research on Diabetes and Associated Metabolic Diseases (CIBERDEM), Instituto de Salud Carlos III, 08907 Barcelona, Spain; josep.franch@gmail.com; 3 Biomedical Research Institute of Lleida, 25198 Lleida, Spain; angels.betriu.bars@gmail.com (A.B.); martahernandez@gmail.com (M.H.); efernandez@irblleida.cat (E.F.); 4 Department of Endocrinology and Nutrition, University Hospital Arnau de Vilanova, 25198 Lleida, Spain; 5 Department of Endocrinology and Nutrition, Institut d’Investigacions Biomèdiques August Pi Suñer, CIBEROBN, Hospital Clinic, 08036 Barcelona, Spain; eortega1@clinic.cat; 6 Department of Medicine, Barcelona Autonomous University (UAB), 08916 Barcelona, Spain; 7 Primary Health Care Center Raval Sud, Gerència d’Atenció Primaria, Institut Català de la Salut, 08001 Barcelona, Spain; 8 Department of Medicine, University of Padova, 35128 Padova, Italy; angelo.avogaro@unipd.it; 9 Department of Endocrinology and Nutrition, University Hospital de la Santa Creu i Sant Pau & Institut d’Investigació Biomèdica Sant Pau (IIB Sant Pau), 08041 Barcelona, Spain

**Keywords:** type 1 diabetes, plaque characteristics, carotid plaque, echogenic plaque

## Abstract

The aim of the study was to investigate ultrasound tissue characterization of carotid plaques in subjects with and without diabetes type 1 (T1D). B-mode carotid ultrasound was performed to assess the presence and type of plaque in a group of 340 subjects with and 304 without T1D, all of them without cardiovascular disease. One hundred and seven patients with T1D (49.5% women; age 54 ± 9.8 years) and 67 control subjects without diabetes who had at least one carotid plaque were included in the study. The proportion of subjects who had only echolucent plaques was reduced in the group of patients with T1D (48.6% vs. 73.1%). In contrast, the proportion with only echogenic (25.2% vs. 7.5%) and calcified plaques (9.4% vs. 1.5%) was increased compared with subjects without diabetes. Moreover, having at least one echogenic plaque was more frequent in T1D patients compared with subjects without diabetes (49.5% vs. 26.9% *p* = 0.005). In addition to diabetes (OR 2.28; *p* = 0.026), age (OR 1.06, *p* = 0.002) was the other variable associated with echogenic plaque existence in multiple regression analysis. Patients with T1D exhibit a differential pattern of carotid plaque type compared with subjects without diabetes, with an increased frequency of echogenic and extensively calcified plaques.

## 1. Introduction

Atherosclerotic cardiovascular disease (CV) is a major cause of mortality in patients with type 1 diabetes [1,2]. Its main clinical manifestations include myocardial infarction, stroke and peripheral vascular disease. The common nexus of this group of cardiovascular events is atherosclerosis, which is characterized as a chronic lipid deposition process in the sub-endothelial space that develops insidiously throughout life and is typically in an advanced stage when its symptoms manifest clinically in the form of a cardiovascular event.

Ultrasonography is a non-invasive, safe, reproducible and well-validated method for visualizing and quantifying atherosclerotic lesions [3]. The presence and burden of subclinical carotid atherosclerotic plaque is strongly associated with future arterial CV events [4,5]. A high prevalence of carotid plaques has been described in patients with type 2 diabetes without evidence of clinical CV disease [6]. Data regarding atherosclerotic plaques in patients with type 1 diabetes are very scarce. In this sense, our group has recently described an increased proportion and burden of carotid plaques in a group of 340 patients with T1D without CV events compared with a group of subjects without diabetes [7]. On the other hand, plaque echogenicity as assessed by carotid ultrasound reliably predicts the content of soft tissue and the amount of calcification in carotid plaques [8]. In this regard, echolucent plaques exhibit more lipid, inflammatory cells and haemorrhage compared with echogenic plaques, which contain more calcification and fibrous tissue; characteristics that have been validated by histopathology [8,9,10]. Otherwise, calcified plaques in the coronary arteries determined by computed tomography scanning coronary artery calcium (CAC) are accepted as a measure of CV disease burden both in patients with [11] and without diabetes [4]. Vascular calcification beyond coronary arteries (i.e., carotid and aortic plaques) has also been described to be associated with all-cause and CV mortality both in patients with [12] and without type 2 diabetes [13].

Studies that have evaluated plaques characteristics by carotid ultrasonography demonstrated that lipid rich plaques (echolucent) are associated with an increased risk of CV events in patients with symptomatic stenotic (≥50%) plaques [14,15]. Most studies that have analyzed the presence and ultrasound characteristics of carotid plaques in patients with diabetes have been performed in asymptomatic subjects with T2D. In these patients both echolucent [16,17] and echogenic [18] plaques were shown to be associated with an increased risk of future CV events. Data regarding subclinical atherosclerotic plaques characteristics assessed by carotid ultrasound in patients with type 1 diabetes are lacking. To our knowledge, no studies to date have analyzed plaque characteristics by carotid ultrasound in patients with type 1 diabetes.

The present study aimed to analyze ultrasound non-stenotic carotid plaque echogenicity in a group of patients with type 1 diabetes and to compare the results obtained with a group of subjects without diabetes, all of whom do not have previous history of clinical CV disease and exhibit normal renal function.

## 2. Experimental Section

### 2.1. Subjects

This study is a sub-analysis of a previous study from our research group [7]. From that study, 174 subjects with at least one atherosclerotic plaque, including 107 patients with type 1 diabetes and 67 without diabetes, were selected. Our previous study was a cross-sectional study conducted in 340 patients with type 1 diabetes and 304 subjects without diabetes matched by sex and age. All participants were free from previous CV disease. Subjects with type 1 diabetes were recruited from the diabetic outpatient clinics at University Hospital Germans Trias i Pujol and University Hospital Arnau de Vilanova in the north-western region of Spain (Catalonia). All potential participants were identified from the electronic clinical records from the two participating institutions that belong to the same health care organization.

For type 1 diabetic subjects, the inclusion criteria were as follows: age >18 years; diabetes for at least 1 year; normal renal function (estimated glomerular filtration rate (eGFR >60 mL/min)); no previous CV disease defined as any form of clinical coronary heart disease, stroke or peripheral vascular disease (including any form of diabetic foot disease). Moreover, we excluded patients with a urine albumin excretion ratio >300 mg/g. The selection of subjects without diabetes was based on the same criteria, except for the criteria that concerned the diabetic specific microvascular complications. Additionally, subjects in the control group had fasting glucose and HbA1c values less than 100 mg/dL and 5.7% (39 mmol/mol), respectively.

For each subject, age, sex, body mass index and waist circumference were obtained by standardized methods. Subjects were considered to have hypertension or dyslipidaemia if they were under anti-hypertensive or lipid-lowering agent treatment, respectively. Serum and spot urine samples were collected in the fasting state, and all serum and urine tests were performed using standard laboratory methods as previously described [19].

### 2.2. Carotid Ultrasound Imaging

The detailed protocol used to evaluate the presence of carotid plaques by ultrasound has been previously described [19]. Briefly, carotid ultrasonography imaging was performed using a LOGIQ^®^ E9 (General Electric, Wauwatosa, WI, USA) equipped with a 15-MHz linear array probe or a Sequoia 512 (Siemens, North Rhine, Westphalia, Germany) equipped with a 15-MHz linear array probe. Plaques were identified using B-mode and colour Doppler examinations in both the longitudinal and transverse planes to consider circumferential asymmetry and were defined as a “focal structure that encroaches into the arterial lumen of at least 0.5 mm or 50% of the surrounding carotid intima media thickness value or demonstrates a thickness of 1.5 mm, as measured from the media-adventitia interference to the intima-lumen surface” according to the Mannheim consensus [20]. Further, the LOGIQ E9 ultrasound system had a 3D image acquisition system with a low depth sweep box with a 45° angle and a slow acquisition time to scan the volume. Volume acquisition used 3 orthogonal sectional plans (A, B, C) and started with the sectional image (A), which gave a 2D image showing the longitudinal view of the carotid arteries and bulb. Images B and C showed the transverse and horizontal axes, respectively. The volume sweep was recorded as raw data and used for volume calculations. Volume calculation was made by delineating the plaque contours and with the transverse plane (B) as described previously [21]. Plaque volume was evaluated in 96 subjects (67 with type 1 diabetes and 29 without diabetes. Volume plaque was measured as total and mean plaque volume. Total plaque volume was defined as the sum of all plaque volume, and mean plaque volume is the sum of the mean plaque volume of each subject.

Atherosclerotic plaques were classified using the well-known five-type classification system based on visual assessment of echogenicity with vessel lumen and adventitia as reference structures: Uniformly echolucent, predominantly echolucent, uniformly echogenic, predominantly echogenic and extensively calcified plaques [19]. We additionally reclassified individuals into 4 clinical categories that have been shown to be useful in terms of future outcomes [16]: Echolucent (lipid- and haemorrhage-rich plaques), mixed (when patients had both echolucent and echo-rich plaques), echogenic (fibrotic or fibro-fatty plaque); and extensively calcified plaques (echo-shadowing from calcifications) as previously described [9]. This classification has been validated against histopathology [9,22] and grey scale medium measured on ultrasound images [23]. The arterial territories explored included the common and internal carotid territories and the bifurcation from the left and right carotid arteries. All participants in the study underwent the same carotid ultrasound examination, and all measures and ultrasound studies were assessed at each participating hospital by the same researcher.

The Local Ethics Committee of both participating centers approved the protocol (PI11/11 and PI-13-095) in accordance with the Declaration of Helsinki, and all participants signed informed consent forms.

### 2.3. Statistical Analysis

The descriptive statistics of the mean (standard deviation) or median [interquartile range] were estimated for quantitative variables with a normal or non-normal distribution, respectively. For the qualitative variables, absolute and relative frequencies were used. Normally distributed data were analyzed using the Shapiro–Wilk test. The differences between groups were assessed by Student’s test, analysis of variance or Mann–Whitney test, and Kruskal–Wallis test depending on the distributions of the quantitative variables. The significance of the differences in qualitative variables was assessed by Chi-squared test or Fisher’s exact test. A conditional logistic regression model was performed to study the echogenic plaque and its association with other variables. All variables of the bivariate analysis with a *p*-value < 0.2 were used. Only the main effects with a significant contribution to the final model according to the likelihood ratio test were included in the final model. Goodness of fit logistic model assumptions were evaluated using the Hosmer–Lemeshow test. Statistical significance was established at a *p*-value < 0.05. Data management and analyses were performed with the free software environment R version 3.3.1 and SPSS software (version 22, SPSS, Chicago, IL, USA).

## 3. Results

Clinical characteristics of the 174 subjects with carotid plaque included in this study are presented in Table 1. Patients with type 1 diabetes exhibited an increased prevalence of hypertension and dyslipidaemia. Among them, up to 60 (56.1%) had diabetic retinopathy. The duration of diabetes was 24.7 (±12) years. In addition, 76 (71%) patients were under statin treatment, and 53 (49.5%) were under antiplatelet treatment. Patients with type 1 diabetes also exhibited reduced waist circumference, diastolic blood pressure, and plasma triglyceride, total cholesterol and low-density lipoprotein (LDL) cholesterol concentrations compared with the non-diabetic group. Moreover, patients with type 1 diabetes exhibited increased systolic blood pressure, heart rate, and high-density lipoprotein (HDL) cholesterol concentrations compared with the group of subjects without diabetes (Table 1).

### 3.1. Ultrasound Examination

**Figure 1:**
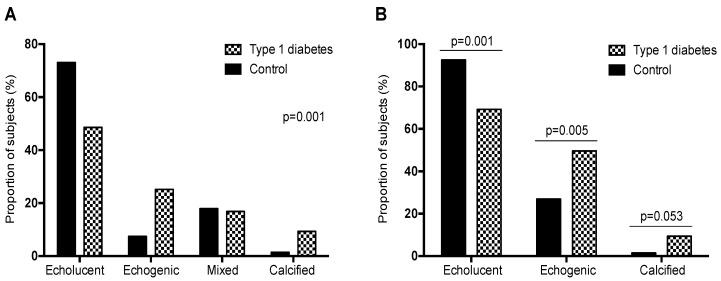
Atherosclerotic plaque type. (**A**) Proportion of patients with different types of atherosclerotic plaques (only echolucent, only echogenic, mixed of echolucent and echogenic plaques, and calcified with or without another plaque type). (**B**) Proportion of patients with different types of atherosclerotic plaques (at least one echolucent plaque, at least one echogenic plaque, and at least one calcified plaque).

No differences were observed in the proportion of subjects with either one or more carotid plaque between patients with type 1 diabetes and subjects without diabetes (Table 1). Regarding plaque echogenicity, differences were observed between study groups (*p* = 0.001) (Figure 1A). The proportion of patients with type 1 diabetes who had only echolucent, i.e., lipid-rich plaques, was reduced in patients with type 1 diabetes compared with subjects without diabetes (48.6% vs. 73.1%, respectively). In contrast, the proportion of patients with only echogenic plaques, i.e., those who contain more calcification and fibrous tissue, was increased in the group of patients with type 1 diabetes (25.2% vs. 7.5%). In addition, the proportion of patients with at least one echogenic plaque was increased in patients with type 1 diabetes compared with subjects without diabetes (49.5% vs. 26.9%, *p* = 0.005) (Figure 1B). When stratifying the groups by sex, differences in the proportion of patients with echogenic plaques were observed. In subjects without diabetes, the proportion of those with echogenic plaques was increased in men (12.8% vs. 0%). In the group of patients with type 1 diabetes no differences were found between sexes (24.1% in men and 26.4% in women).

Finally, no differences were noted in the proportion of patients with mixed plaque types (echolucent and echogenic) between the study groups (16.8% vs. 17.9%). Additionally, the proportion of patients with at least one extensively calcified plaque was increased in patients with type 1 diabetes compared with subjects without diabetes (9.4% vs. 1.5%) (Figure 1B). None of the study subjects had high-grade (>50%) carotid artery stenosis.

### 3.2. Measurements of the Plaque Volume

Plaque volume was assessed in 96 subjects; 67 type 1 diabetes patients and 29 control subjects. There were no differences of total plaque volume between subjects with and without diabetes, median [IQR], (60 [30; 130] mm^3^ vs. 50 [20; 80] mm^3^; *p* = 0.260). However, the mean plaque volume was higher in type 1 diabetes patients compared to control subjects (40 [30; 70] mm^3^ vs. 30 [20; 40] mm^3^; *p* = 0.028). On the other hand, plaque volume (total and mean) were higher in individuals with echogenic plaque compared with individuals with non-echogenic plaque 100 [60; 160] mm^3^ vs. 40 [20; 69] mm^3^; *p* < 0.001 and 55 [34.2; 80] mm^3^ vs. 30 [20; 50] mm^3^; *p* = 0.003, respectively).

### 3.3. Characteristics of Patients with Echogenic Plaques

Seventy-one study subjects exhibited echogenic plaques (including those with or without type 1 diabetes). Among them, up to 53 (74.6%) had type 1 diabetes and up to 41 (57.7%) were under statin treatment. Subjects with echogenic plaques were older, exhibited an increased frequency of hypertension and dyslipidaemia, had higher systolic blood pressure and were more frequently under antiplatelet treatment. On the other hand, total and LDL cholesterol concentrations were reduced in those patients with echogenic plaques compared with the group without echogenic plaques. Finally, the proportion of patients with more than one plaque was increased in the group of subjects with echogenic plaques (Table A1 in Appendix A).

### 3.4. Logistic Regression Model of Echogenic Plaques

The multiple regression model was constructed using the candidate parameters identified in the bivariate analysis and the clinically relevant variables to explain the risk of having echogenic plaques. The model for having echogenic plaques vs. not having echogenic plaques revealed that the variables associated with the presence of echogenic plaques were older age (OR = 1.06; *p* = 0.002) and type 1 diabetes (OR = 2.28; *p* = 0.026); this model had a good discriminatory ability (AUCROC = 0.73; *p* < 0.001) (Table 2).

## 4. Discussion

In the present study that included a large sample of patients with type 1 diabetes and subjects without diabetes, all of whom had at least one carotid atherosclerotic plaque, we report for the first time that the ultrasound tissue characterization of carotid plaques differs between patients with type 1 diabetes and subjects without diabetes. The results obtained show that the proportion of subjects with echogenic or extensively calcified plaques is increased in patients with type 1 diabetes compared with subjects without diabetes, whereas the proportion of echolucent plaques is lower in the former.

In the general population, non-invasive measures of subclinical atherosclerosis, such as CAC burden and atherosclerotic plaque presence and burden in the carotid arteries, are features associated with an increased risk of CV events [4,8,12,13]. In addition, plaque characteristics are associated with the future risk of CV events. Regarding carotid plaques, ultrasonographic plaque characterization studies performed in the general population have demonstrated that echolucent plaques are independently associated with an increased risk of cerebrovascular and other CV events [24] in symptomatic patients with stenotic plaques (≥50%) [15] and those with non-stenotic plaques [25]. On the other hand, plaque volume, as well as the number of atherosclerotic plaques (i.e., plaque burden), have both been associated with an increased risk of future CV events [26]. First, in our study, the mean plaque volume in type 1 diabetic subjects was higher, although this preliminary finding should be interpreted with caution as the number of subjects is quite low. This prevented us from further statistical work-up using these data. Interestingly, the proportion of patients with more than one plaque was increased in the group of subjects with echogenic plaques. In concordance with this finding, these patients had a higher total plaque volume than the non-echogenic group. Thus, the presence of echogenic plaques in a given patient is associated with an increased burden of atherosclerosis (total number and volume of plaques). Therefore, future studies should address the issue of whether subjects with echogenic plaques, especially if they have type 1 diabetes, are at a higher risk of cardiovascular events than those with echolucent plaques. Additionally, in the general population, carotid plaque calcification, which is defined as that with an acoustic shadowing, is a strong and an independent predictor of vascular events in asymptomatic subjects [11,27]. In type 2 diabetes, the presence of both echolucent [16,17,28] and echogenic plaques [12,18] is also independently associated with an increased risk of CV events. In contrast to echolucent plaques, which are lipid rich, echogenic plaques contain more calcification and fibrous tissue. In the general population and patients with type 2 diabetes carotid plaque calcification, as assessed by computed tomography, is independently associated with a future risk of cardiovascular events [12]. Not only the presence of calcium in the atherosclerotic plaque but also the pattern of the calcium depots has been suggested to be involved in the vulnerability of the atherosclerotic lesion. In this sense, microcalcification patterns, i.e., spotty calcium depots, are associated with vulnerability of coronary atherosclerotic plaques in contrast to extensive calcifications, which are associated with stable plaques [29,30,31]. These findings suggest that microcalcification may increase wall stress in the fibrous cap, resulting in plaque instability as a characteristic of vulnerable plaques [31,32]. In the present study, presence and characteristics of carotid plaques were evaluated with carotid B-mode ultrasound. Actually, as contrast-enhanced modalities that are the best method to analyze the characteristics of vulnerable atherosclerotic plaques such as inflammation, intraplaque haemorrhage and ulceration, the use of this ultrasound method might have clearly improved the adequate characterization of carotid atherosclerotic plaques of type 1 diabetic patients; unfortunately, this is a rather time-consuming and expensive procedure that we could not use in the current study.

Few studies have analyzed the presence and tissue characteristics of atherosclerotic plaques in type 1 diabetes. Atherosclerotic CV disease is the leading cause of death in people with type 1 diabetes whose risk of CV disease is two- to seven-fold increased and not completely accounted for by traditional CV risk factors [33]. For example, women exhibit a two-fold increased risk of fatal and nonfatal vascular events compared with men with type 1 diabetes [1]. Our group has recently described an increased proportion and burden of carotid plaques in type 1 diabetic patients without CV events compared with subjects without diabetes [7]. In this group of patients, the presence of advanced stages of diabetic retinopathy is independently associated with the presence and the burden of subclinical carotid atherosclerosis. Plaque tissue characteristics in patients with type 1 diabetes have been mainly studied in coronary arteries by computed tomography. Studies demonstrate that diabetes is associated with an increased prevalence of any degree of calcification compared with control subjects [32]. Moreover, coronary artery calcification is greatly increased in women, and the gender difference in calcification observed in the general population is lost in subjects with type 1 diabetes [34,35]. In addition, Djaberi et al., used multislice computed tomography to assess coronary atherosclerosis and found no differences in average CAC scores and the prevalence of coronary atherosclerosis between asymptomatic patients with type 1 and type 2 diabetes, with the exception of an increased percentage of noncalcified plaques in patients with type 2 diabetes [36].

To the best of our knowledge, no studies to date have analyzed the ultrasound characteristics of carotid plaques in patients with type 1 diabetes. The results obtained in the present study demonstrated that the proportion of subjects with echogenic plaques is increased in patients with type 1 diabetes compared with subjects without diabetes without differences between gender in the proportion of subjects who have echogenic plaques. These results point to a greater effect of calcification in women compared with men given that echogenic plaques contain more calcified tissue compared with echolucent plaques. These results are similar to those that have described a reduction in the gender difference in coronary artery calcification given that the prevalence of CAC is similar in men and women [35]. The analysis performed in the present study to analyze those variables associated with the risk of having echogenic plaques noted that older age and type 1 diabetes were the only ones that were independently associated with the risk of having this type of plaque. However, we must acknowledge that although we found an association between each of these two variables with the presence of echogenic plaques, the low number of subjects studied may reduce the chance of finding an association with other cardiovascular disease-related or unexplored factors. Finally, regarding extensive plaque calcification, our results demonstrate that the proportion of patients with at least one extensively calcified plaque was increased in patients with type 1 diabetes compared with subjects without diabetes, which is similar to the increased coronary artery calcification described in patients with type 1 diabetes compared with nondiabetic subjects.

**Figure A1:**
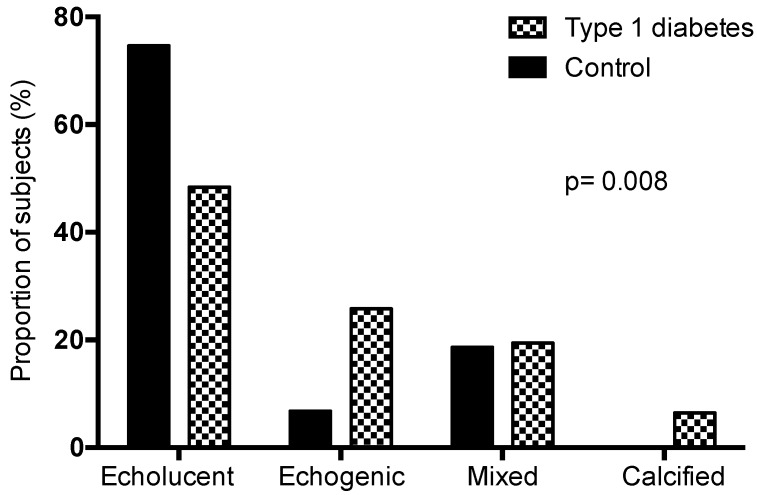
Proportion of patients without statin treatment with different types of atherosclerotic plaque (only echolucent, only echogenic, mixed of echolucent and echogenic plaques, and calcified with or without another plaque type), *n* = 90.

In the general population, treatment with statins promotes coronary artery calcification [37]. Few studies have analyzed the effect of statin treatment on calcium content in coronary arteries in patients with diabetes mellitus. These studies have been performed in patients with type 2 diabetes. The results indicate that patients who receive statins exhibit an increased degree of calcification as assessed by CAC compared with those who are not receiving statins; however, the progression of CAC scores is faster in those who are not receiving statins [38,39]. In this sense, some authors have suggested that statins render microcalcifications more confluent, which could be associated with a reduction in vessel wall stress [40]. In fact, patients with diabetes and patients with a previous CV event are frequently treated with statins given that this treatment decreases the incidence of CV events in these populations. Our study reported no differences in the plaque type distribution between the complete series and patients without statin treatment (Figure A1 in Appendix A), or between subjects under statin treatment with and without echogenic plaques.

Our study has several limitations. First, although we adjusted for risk factors known to be associated with atherosclerosis, it is possible that some confounding factors exist and were incompletely accounted for. Second, the cross-sectional design precludes conclusions about causality; therefore, a larger prospective study is needed to establish the usefulness of plaque type characterization as a predictive factor for CV events in patients with type 1 diabetes. Finally, an additional limitation is that only a limited number of patients with echogenic plaques were studied; therefore, larger studies are warranted as no final conclusions can be drawn from our findings.

## 5. Conclusions

In summary, our study demonstrates that in the absence of previous CV disease, non-stenotic carotid plaque ultrasound characteristics differ between patients with type 1 diabetes and subjects without diabetes. The proportion of echogenic plaques, which comprise calcified tissue, is increased in patients with type 1 diabetes, and no differences exist in the proportion of subjects with echogenic plaques between genders. This finding is consistent with the notion that diabetes is associated with vascular calcification and the loss of gender differences in calcification. Whether the increase in the proportion of plaques with this ultrasonographic pattern observed in patients with type 1 diabetes will translate into an increased risk of future cardiovascular events will be determined in the prospective follow-up of this cohort of patients.

## Figures and Tables

**Table 1:** Baseline characteristics of the participants.

	Non-Diabetic Group (*n* = 67)	Type 1 Diabetes (*n* = 107)	*p*-Value
Age, years	52.6 (9.13)	54 (9.8)	0.351
Sex, women, *n* (%)	28 (41.8%)	53 (49.5%)	0.401
Race, non-Caucasian, *n* (%)	2 (3%)	0 (0%)	0.147
Tobacco exposure, *n* (%)	35 (53%)	70 (65.4%)	0.144
Antiplatelet treatment, *n* (%)	3 (10.3%)	53 (49.5%)	<0.001
Dyslipidaemia, *n* (%)	20 (29.9%)	78 (72.9%)	<0.001
Hypertension, *n* (%)	13 (19.4%)	60 (56.1%)	<0.001
Systolic blood pressure, mmHg	131 (14.2)	137 (18)	0.010
Diastolic blood pressure, mmHg	79.6 (8)	74.7 (11.6)	0.001
Statin treatment, *n* (%)	8 (11.9%)	76 (71%)	<0.001
Heart rate, beats/min	71 (11.1)	76.8 (11.9)	0.002
Body mass index, kg/m^2^	27.8 [25; 30.3]	26.4 [24.3; 29.6]	0.124
Waist, cm	99.6 (12.8)	92.9 (12.7)	0.001
HbA1c, %	5.5 [5.3; 5.7]	7.6 [7.15; 8.15]	<0.001
HbA1c, mmol/mol	36.6 [34; 39]	60 [54; 65.5]	<0.001
Total cholesterol, mg/dL	203 [185; 228]	173 [155; 198]	<0.001
HDL cholesterol, mg/dL	54 [46; 63]	59 [52; 70]	0.005
LDL cholesterol, mg/dL	124 [112; 148]	97.4 [80.6; 113]	<0.001
Triglycerides, mg/dL	99 [65; 142]	71 [57; 90]	0.001
Creatinine, mg/dL	0.81 [0.7; 0.9]	0.79 [0.7; 0.9]	0.166
GFR, mL/min/1.73 m^2^	97.9 [85.6; 105]	96.1 [88.3; 105]	0.899
Diabetes duration, years	-	24.7 (12)	-
Plaque			0.581
One plaque	35 (52.2%)	50 (46.7%)	
Multiple plaques (≥2)	32 (47.8%)	57 (53.3%)	

Data are mean (SD) or median [interquartile range] or *n* (%). GFR, Glomerular filtration rate estimate based on the CKD-EPI (Chronic Kidney Disease Epidemiology Collaboration) equation; HDL, high-density lipoprotein; HDL, low-density lipoprotein.

**Table 2:** Logistic regression model for the presence of any echogenic plaques.

Presence of any Echogenic Plaque vs. No Echogenic Plaque ^a^			
	Odds Ratio	95% CI	*p*
Age	1.062	1.023–1.103	0.002
Sex, women	0.539	0.272–1.068	0.076
BMI	0.949	0.872–1.032	0.221
Diabetes	2.276	1.104–4.690	0.026
sBP	1.019	0.998–1.040	0.075

BMI, body mass index; sBP, systolic blood pressure. ^a^ The logistic model showed a good discrimination with an AUC of 0.73 (95% CI: [0.65–0.80] and no significant lack of calibration (Hosmer–Lemeshow test *p*-value = 0.21).

## Appendix A

**Table A1:** Baseline characteristics of the study group according to the presence of echogenic plaques.

Variable	Non-Echogenic Plaque	Echogenic	*p*-Value Plaque
	*n* = 103	*n* = 71	
Age, years	51.2 (9.22)	56.7 (9.11)	<0.001
Sex, women, *n* (%)	52 (50.5%)	29 (40.8%)	0.272
Race, non Caucasian, *n* (%)	2 (1.94%)	0 (0.00%)	0.514
Diabetes mellitus type 1, *n* (%)	54 (52.4%)	53 (74.6%)	0.005
Diabetes duration, years	22.2 (11.8)	27.2 (11.8)	0.030
Tobacco exposure, *n* (%)	58 (56.9%)	47 (66.2%)	0.281
Hypertension, *n* (%)	33 (32.0%)	40 (56.3%)	0.002
Dyslipidaemia, *n* (%)	47 (45.6%)	43 (60.6%)	0.075
BMI, kg/m^2^	27 [25;29.9]	26.7 [24.2;29.8]	0.424
Waist, cm	96.0 (12.0)	94.5 (14.6)	0.469
Systolic blood pressure, mmHg	132 (17.4)	139 (15.5)	0.004
Diastolic blood pressure, mmHg	76.3 (10.2)	76.9 (11.3)	0.730
Heart rate, beat/min	74.1 (12.8)	75.6 (10.6)	0.391
Antiplatelet treatment, *n* (%)	23 (29.1%)	33 (57.9%)	0.001
Statin treatment, *n* (%)	43 (41.7%)	41 (57.7%)	0.055
Glucose, mg/dL	99 [87.5; 140]	127 [88; 211]	0.034
HbA1c, %	6.7 [5.5; 7.6]	7.40 [6.3; 7.9]	0.006
HbA1c, mmol/mol	49.7 [36.6; 60]	57 [45; 63]	0.006
Triglycerides, mg/dL	74 [59; 106]	81 [60.5; 126]	0.313
Total cholesterol, mg/dL	192 [170; 218]	178 [160; 202]	0.016
HDL, mg/dL	58 [49.5; 70]	56 [47.5; 68.5]	0.296
LDL, mg/dL	112 [92.5; 141]	107 [82.5; 120]	0.036
Creatinine, mg/dL	0.8 [0.67; 0.9]	0.79 [0.66; 0.92]	0.756
GFR, mL/min/1.73 m^2^	97.6 [87.4; 106]	96 [86.8; 104]	0.485
Plaque, *n* (%)			<0.001
One plaque	67 (65%)	18 (25.4%)	
Multiple plaques	36 (35%)	53 (74.6%)	

Data are mean (SD) or median [interquartile range] or *n* (%). GFR, Glomerular filtration rate estimate by the CKD-EPI (Chronic Kidney Disease Epidemiology Collaboration) equation.
